# High-risk oncogenic HPV genotypes in vulnerable women from the Amazon: a cross-sectional retrospective study

**DOI:** 10.1186/s12985-026-03220-3

**Published:** 2026-06-11

**Authors:** Darciane Coelho Cordovil, Laryssa Danielle da Silva Reis, Lumara Silvia Santana Ferreira, Carina Guilhon Sequeira, Thiago de Matos Bezerra, Edivaldo Herculano Correa de Oliveira, Rogério Valois Laurentino, Luiz Fernando Almeida Machado, Leonardo Miranda dos Santos, Ana Maria Revoredo da Silva Ventura, Rodrigo Vellasco Duarte Silvestre

**Affiliations:** 1 Postgraduate Program in Epidemiology and Health Surveillance, Virology Section, Evandro Chagas Institute, Ananindeua, Brazil; 2 Retrovirus and Papillomavirus Laboratory, Virology Section, Evandro Chagas Institute, Ananindeua, Brazil; 3 Laboratory of Molecular and Cellular Biology, Tropical Medicine Center, Federal University of Pará, Belém Pará, Brazil; 4 Laboratory of Cytogenetics and Environmental Mutagenesis, Environment Section, Evandro Chagas Institute, Ananindeua, Brazil; 5 Virology Laboratory, Institute of Biological Sciences, Federal University of Pará, Belém, Brazil

**Keywords:** Human Papillomavirus (HPV), Amazônia, Public Health, Genotyping, Cervical Cancer

## Abstract

**Introduction:**

Human papillomavirus (HPV) is widely acknowledged as the primary etiological agent of cervical cancer, with more than 14 high-risk oncogenic genotypes identified to date. Women residing in remote and underserved regions of the Amazon face significant barriers to accessing quality healthcare services, including geographic isolation, socioeconomic disparities, and limited infrastructure. These challenges contribute to the disproportionate burden of cervical cancer observed in this population, where women from interior regions represent the highest percentage of cases in Brazil.

**Objective:**

This study aims to elucidate the prevalence, distribution of HPV genotypes, and associated risk factors among women from remote communities in the Brazilian Amazon, providing critical insights into regional public health challenges.

**Methods:**

Endocervical smears were systematically collected from women across 18 municipalities. HPV detection was performed using Hybrid Capture II, a highly sensitive molecular assay, while genotyping was conducted using the Linear Array HPV system, which enables comprehensive identification of multiple HPV genotypes. Statistical analyses included chi-square tests, odds ratios (OR), and Cramér’s V, with a significance threshold set at *p* ≤ 0.05 and a 95% confidence interval.

**Results:**

The overall prevalence of HPV infection was 13.84% (182/1315), with notable variation across municipalities. Irituia exhibited the highest prevalence at 21.25% (*p* = 0.0664). Key factors associated with HPV acquisition included age ≤ 25 years (*p* < 0.0001), unmarried status (*p* < 0.0001), and non-use of condoms (*p* = 0.0032). Fifteen different genotypes of HPV were found. Of the total positive samples, only 58.8% (*n* = 107) samples were genotyped. High-risk oncogenic HPV was detected in 63 genotyped samples (58.9%), the most frequent high-risk oncogenic HPV genotypes were HPV16 (*n* = 15, 14%), HPV59 (*n* = 10, 9.3%), and HPV58 (*n* = 9, 8.4%). Co-infections involving both high- and low-risk genotypes were observed in 14% (8/57) of the samples, while mixed infections accounted for 22.8% (13/57).

**Conclusion:**

Our findings highlight the significant burden of HPV infection among young, unmarried women who do not use condoms, emphasizing the urgent need for targeted interventions. The low quality of the DNA obtained prevented us from genotyping all samples. The high diversity of high-risk HPV genotypes, particularly the predominance of HPV16 in all municipalities studied, reveals a potential critical vulnerability in this population. These results emphasize the importance of strengthening screening programs, promoting condom use, and implementing vaccination campaigns to mitigate the spread of HPV and reduce the incidence of cervical cancer in the Brazilian Amazon.

**Supplementary Information:**

The online version contains supplementary material available at 10.1186/s12985-026-03220-3.

## Introduction

Human papillomavirus (HPV) is a DNA virus that belongs to the Papillomaviridae family and is characterized by its ability to infect the skin and mucous membranes of vertebrates. Based on the similarity profile of the *L1 gene*, more than 200 distinct genotypes of HPV have been identified in vertebrate infections and are classified into five specific groups (α, β, γ, µ, and ν) [1]. Cervical Cancer (CC) ranks as the fourth leading cause of death among adult women globally, with approximately 660,000 new cases and 350,000 deaths reported annually, predominantly in low- and middle-income countries [2]. Globally, the prevalence of HPV infection is estimated at 31%, with 21% attributed to high-risk HPV genotypes among young adults aged 25 to 29 years [3]. Cervical cancer remains a significant global public health challenge, with approximately 90% of cases diagnosed in underdeveloped and developing countries [4]. In Brazil, cervical cancer has an incidence rate of 15.38 cases per 100,000 women and more than 6,000 deaths annually; however, in the Amazon region in the North of the country, this rate is surprisingly higher [5], directly linked to the high incidence of high-risk oncogenic HPV genotypes, such as HPV16 and HPV18, which are present in approximately 70% of invasive cervical cancer cases. Other HPV genotypes, including HPV31, 33, 35, 39, 45, 51, 52, 56, 58, and 59, are also strongly associated with these cases [6].

Sexually transmitted infections (STIs) caused by HPV can be self-limiting or persistent, resulting in a spectrum of clinical conditions ranging from asymptomatic infections to cytological abnormalities of varying severity in the cervix. These range from abnormalities and cellular lesions of varying degrees to cervical carcinoma in situ and invasive cervical carcinoma [7, 8]. Brazil has a universal health system that proposes a comprehensive and decentralized policy for the prevention of HPV and cervical cancer through primary measures such as a single-dose vaccination schedule and preventive health education measures, and secondary measures offering Pap smear tests as a suggestive diagnostic tool, but it is impossible to detect asymptomatic infection [9]. Molecular biology-based screening for HPV viral DNA, followed by genotyping of specific HPV genotypes, has recently been introduced in the Brazilian Unified Health System (SUS). Although promising, these technologies remain limited to large urban centers and are notably absent from primary healthcare services in remote and peripheral regions of Brazil [10, 11].

The rural populations of the Amazon are characterized by significant socioeconomic vulnerability and limited access to diagnostic opportunities for this and other infections [12, 13]. Many municipalities are located in remote and isolated regions with difficult access, facing substantial logistical, methodological, and even political-ideological challenges that hinder, and in some cases prevent, the implementation of these preventive programs [14, 15, 16]. In Brazil, the overall prevalence of HPV in the general population is approximately 25.4% [17]. HPV prevalence rates in the Amazon vary from 15.5% to 63.3% in specific subpopulations [18–21]. Historically, female populations in the Brazilian Amazon have presented the highest national incidence rates of cervical cancer and face low HPV vaccination coverage [12, 22, 23]. Understanding the distribution of HPV genotypes is crucial for understanding the epidemiological patterns of this infection and provides valuable information revealing the potential impacts of public health policies in this part of the Brazilian Amazon region. The objective of this study was to describe the prevalence, HPV genotypic distribution, and cytological and socioepidemiological factors among sexually active women from remote areas in several municipalities of the Brazilian Amazon region.

## Methods

### Type and study variables

This is a retrospective, cross-sectional, and descriptive study that included a spontaneous population of 1,315 women who voluntarily sought gynecological care in the capital city of Belém, Pará, due to the lack of such care in public services in their places of origin, and underwent routine screening for cervical cancer at the Marco Basic Health Unit, linked to the State University of Pará, in Belém, Amazon region of Brazil, between March 2013 and April 2017. All women aged 18 years or older who had initiated sexual activity were eligible for inclusion. Women were excluded if they were menstruating, pregnant, had undergone partial or total hysterectomy, had a history of severe cervical lesions, or declined to participate in the study and/or did not sign the Informed Consent Form (ICF). The study variables included: place of origin, including the capital city of Belém and several municipalities in the interior (Almerim, Ananindeua, Anajás, Benevides, Colares, Curuçá, Goianésia do Pará, Mosqueiro District, Irituia, Mãe do Rio, Marapanim, Marituba, Salvaterra, Santa Bárbara do Pará, São Miguel do Guamá, and Vigia); age; education level (less than eight years of schooling vs. eight or more years of schooling); alcohol use; tobacco use; condom use; contraceptive use; lifetime number of sexual partners; HPV vaccination status; and cervical-vaginal cytology results in Normal, Atypical Squamous Cells of Undetermined Significance (ASCUS) / Low-grade Squamous Intraepithelial Lesion (LSIL), High-grade Squamous Intraepithelial Lesion (HSIL), Carcinoma in situ, and Invasive Carcinoma). All women who are part of the population of this study, both from the capital and from the interior, were passively recruited and, being a population of spontaneous origin, do not correspond to the sample size of their municipalities of origin, as they voluntarily sought the gynecological services offered in this project through a special initiative.

### Detection of HPV DNA, viral typing, and cytological examination

The biological samples consisted of cells collected from uterine cervical smears using the Digene HC2 DNA Collection Device (Germantown, USA), selected for its ease of use and ability to preserve sample integrity, ensuring high-quality transport for laboratory analysis in accordance with the manufacturer’s recommendations. HPV detection was performed using an initial screening method, the Hybrid Capture II (HC II) assay (Qiagen Gaithersburg, MD, USA), a technique based on signal amplification using RNA probes specific for HPV DNA. A hybridized DNA/RNA complex is formed, and its detection is proportional to the amount of HPV DNA, enabling both qualitative and quantitative screening of each sample. The Hybrid Capture II test was used as an initial screening before genotyping.

HPV infection positivity was determined based on positive results from both the Hybrid Capture II test and the Linear Array HPV Genotyping Test (Roche, Mannheim, Germany). The latter is a technique capable of detecting and typing up to 37 distinct HPV genotypes based on PCR amplification of a segment of the *L1* gene in the HPV viral genome. Subsequently, the amplicon was hybridized on a nylon strip with probes specific for each HPV genotypes, including HPV6, 11, 16, 18, 26, 31, 33, 35, 39, 40, 42, 45, 51, 52, 53, 54, 55, 56, 58, 59, 61, 62, 64, 66, 67, 68, 69, 70, 71, 72, 73, 81, 82, 83, 84, IS39, and CP6108. Coinfection was defined as the simultaneous presence of high- and low-risk HPV genotypes, while multiple infections were identified when more than one distinct HPV type was detected in a single sample. Cytological evaluation was performed according to the Brazilian Nomenclature for Cervical Cytopathology Reporting, adapted from the Bethesda System. The biological samples were deposited in the biorepository of the Papillomavirus Laboratory at the Virology Section of the Evandro Chagas Institute for further investigation. Co-infection is defined as infection with up to two genotypes of HPV, while multiple infections are defined as infection with more than two different genotypes of HPV. All participants who tested positive for sexually transmitted HPV infection were invited for medical evaluation with the medical team at the Virology outpatient clinic of the Evandro Chagas Institute.

### Ethical aspects

The investigations conducted in this study were performed in accordance with the Declaration of Helsinki (1975, revised and updated in 2013), specifically adhering to point 23 of this declaration, and in compliance with the guidelines established by Resolution 466/2012 of the Brazilian National Health Council, Ministry of Health [24]. All 1,315 women who voluntarily attended the health unit were invited to participate in the study. After agreeing to participate, they were fully informed about all stages of the study, including its objectives and justification, and their participation was confirmed upon reading and signing the Informed Consent Form (ICF).

Subsequently, the study participants were invited to complete a questionnaire that included questions addressing socioepidemiological and gynecological variables relevant to this study. Our research is part of the project entitled “Prospective Study to Evaluate the Association Between Molecular Testing for HPV and Cytological Examination (Papanicolaou Test) in the Screening of Uterine Cervical Carcinoma,” which was approved by the Research Ethics Committee of the Evandro Chagas Institute, under the registration number 59,511/CAAE 01977312.4.0000.0019. All data were analyzed while ensuring complete anonymity.

### Statistical analysis

Statistical analyses were performed using SPSS version 21.0 (SPSS, Chicago, IL, USA). For categorical variables with two categories, the test Odds Ratio (OR) were used. For variables with more than two categories, the G-test of Independence was applied. To assess the effect size of these variables, Cramér’s V test was used, with the strength of association classified as weak (≤ 0.29), moderate (0.30–0.49), or strong (≥ 0.50). Logistic regression was performed to avoid potential confounders. A 95% confidence interval (CI) and a significance level of *p* ≤ 0.05 were considered for all analyses.

## Results

### Population characteristics

The median age in the general population was 38 years (SD: 9.5; range: 18–65; IQR: 31.5–46). Among the participants, 90% were aged over 25 years, 73.5% resided in the capital city, and 63.1% had more than eight years of formal education. Regarding marital status, 64.5% were married or in a stable union. Behavioral characteristics revealed that 68.8% did not consume alcohol, 85.9% did not use tobacco, and 72.1% reported using condoms during sexual intercourse. Additionally, 82.1% did not use contraceptive methods, and 67.4% reported having only one lifetime sexual partner. Vaccination coverage was low, with 96.4% having not received the HPV vaccine. Cytological examination results showed that 94.9% exhibited normal findings.

### HPV prevalence

**Fig. 1:**
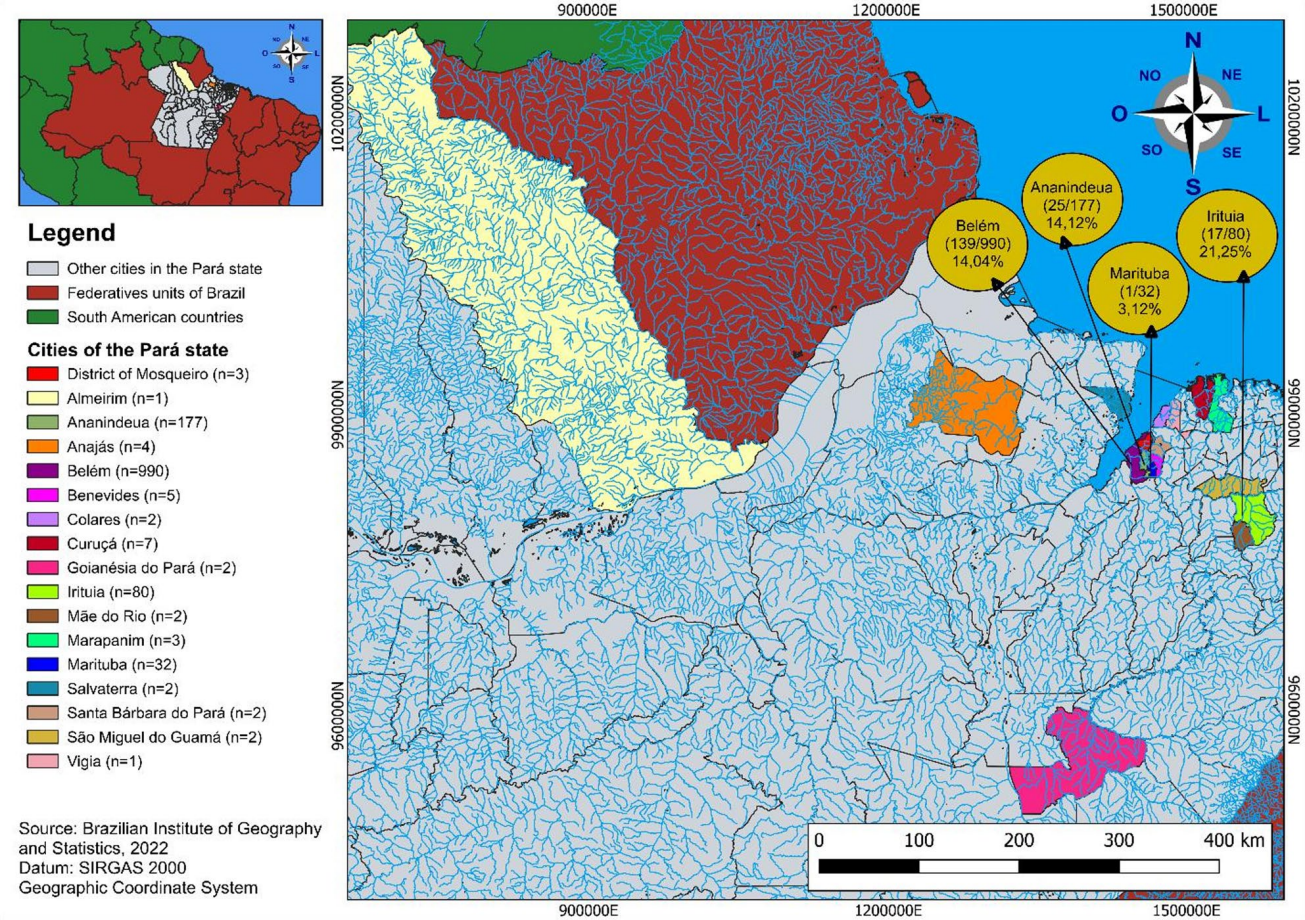
Municipalities of origin and prevalence of HPV-related STIs in women in Amazonian regions

The overall prevalence of HPV infection was found to be 13.84% (182/1,315). The highest prevalence of HPV infection was observed in Irituia (21.25% [17/80]), followed by Belém the Pará State Capital (14.1% [139/990]), Ananindeua (14.12% [25/177]), and Marituba (3.2% [1/32]). Figure 1.

### HPV genotyping

**Fig. 2:**
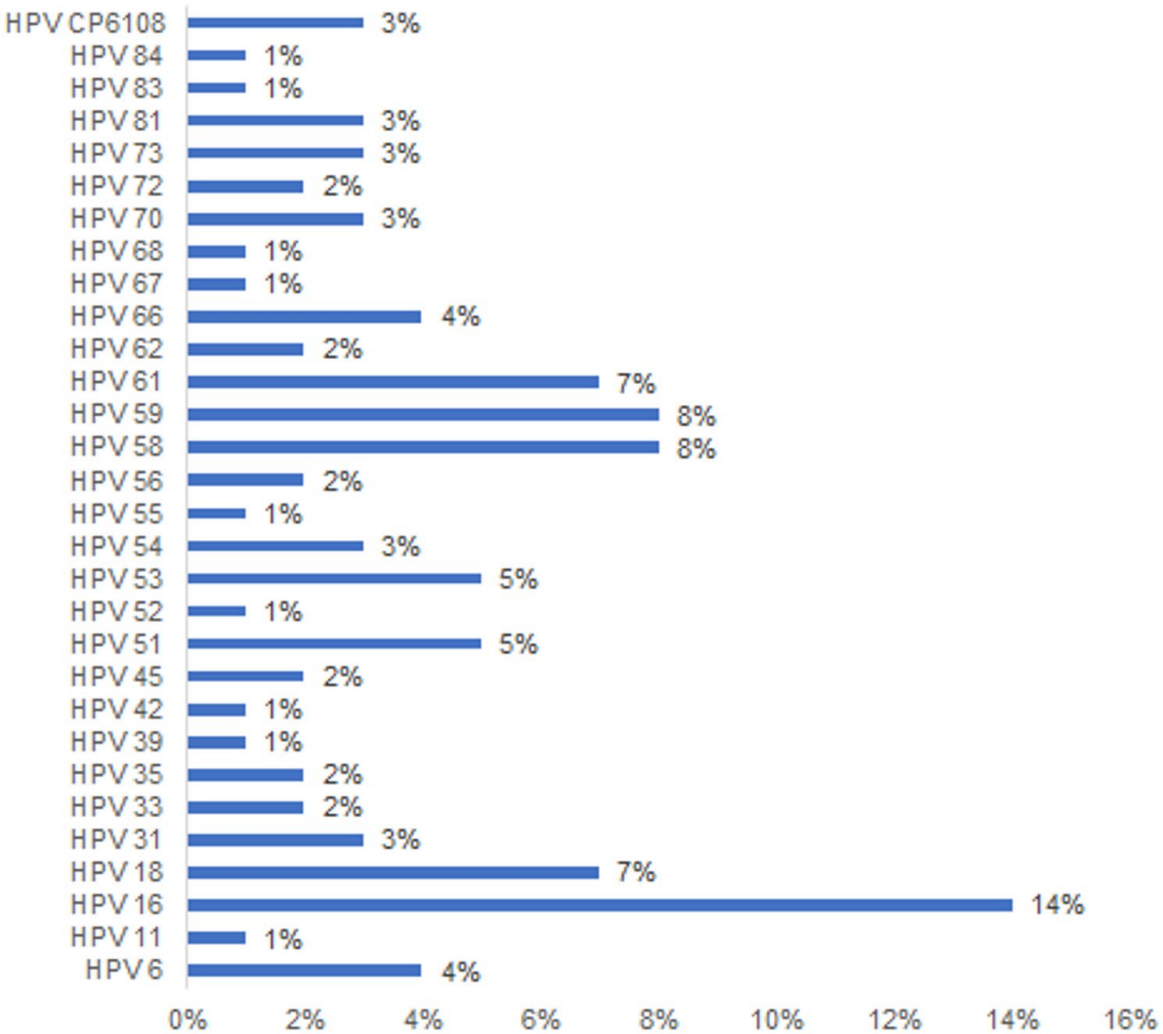
Total frequency of HPV genotypes identified in vulnerable women from the state of Pará, Amazon, Brazil

One hundred and seven samples were genotyped (58.8% of the total). We identify 37 HPV types, the most frequent HPV genotypes were: HPV 16 (*n* = 15 [14%]), HPV 59 (*n* = 10 [9.3%]), HPV 58 (*n* = 9 [8.4%]) and HPV 18 and HPV 61 (*n* = 8 [7.5%] each). Details on HPV genotypes are in Fig. 2 and Supplementary material 1). Coinfections and multiple infections were identified in sixteen and five cases respectively of the total samples (1,279), with oncogenic genotypes present in all cases (Supplementary material 3).

Regarding the geographic distribution of high-risk genotypes, the highest frequency was observed in the Capital city Belém (79.3%, *n* = 50), followed by Ananindeua (9.5%, *n* = 6), Irituia (7.9%, *n* = 5), and Bragança (1.6%, *n* = 1) (Supplementary material 2).

### Risk factors

Among the positive samples, the median age was 33.5 years (SD: 10.15; range: 18–63; IQR: 26–42). Women aged ≤ 25 years were three times more likely to acquire HPV-related STIs compared to those older than 25 years (30.8% [OR: 3.2902, CI: 2.1881–4.9474], *p* < 0.0001). Similarly, unmarried women were nearly twice as likely to acquire HPV-related STIs (19.2% [OR: 1.9539, CI: 1.4249–2.6794], *p* < 0.0001). The lack of condom use nearly doubled the likelihood of acquiring HPV-related STIs (18.5% [OR: 1.6563, CI: 1.1931–2.2993], *p* = 0.0032). There was a trend toward an increased likelihood of acquiring this infection among women who reported alcohol use (16.6% [OR: 1.3796, CI: 0.9950–1.9113], *p* = 0.0537). All associations from Cramer’s V test were weak (Table 1).

**Table 1:** Prevalence, Socioepidemiological and Gynecological Characteristics of Vulnerable Young Women in Multiple Municipalities of the State of Pará, Brazilian Amazon

Characteristics		Total (*n* = 1,315)		HPV+ (*n* = 182)		*p*-value†	Crammer’s V	Odds Ratio	(95% CI)	*p*-value±
		*N*	%	*N*	%					
Age (years)	≤ 25	133	10	41	30.8	< 0.0001*		3.2902	2.1881–4.9474	< 0.0.001
	> 25	1.182	90	141	11.9					
Municipalities of origin	Capital	967	73.5	138	14.3	0.4468		1.1709	0.8139–1.6845	0.990
	Rural	348	26.5	44	12.6					
Educational level (anos)	≤ 8	485	36.9	71	14.6	0.5765		1.1109	0.8055–1.5321	0.142
	> 8	830	63.1	111	13.4					
Marital Status	Single	468	35.5	90	19.2	< 0.0001*		1.9539	1.4249–2.6794	0.004
	Married	847	64.5	92	10.9					
Alcohol use	Yes	410	31.2	68	16.6	0.0537		1.3796	0.9950–1.9113	0.072
	No	905	68.8	114	12.6					
Tobacco use	Yes	185	14.1	19	10.3	0.1609		0.6790	0.4107–1.1227	0.147
	No	1.130	85.9	163	14.4					
Condom use	No	368	27.9	68	18.5	0.0032*		1.6563	1.1931–2.2993	0.025
	Yes	947	72.1	114	12					
Contraceptive Use	Yes	235	17.9	36	15.3	0.5351		1.1573	0.7792–1.7188	0.648
	No	1.080	82.1	146	13.5					
Lifetime number of sexual partners	1	887	67.4	116	13.1	0.4529	0.034			0.634
	2–5	295	22.4	48	16.3					
	> 5	133	10.2	18	13.5					
HPV vaccination	No	1.268	96.4	172	13.6	0.1976		1.7222	0.8410–3.5269	0.181
	Yes	47	3.6	10	21.2					
Cervicovaginal cytology	Normal	1.249	94.9	174	13.9	1.000	0.028			0.360
	ASCUS/LSIL	64	4.8	8	12.5					
	HSIL	0	-	0	-					
	Carcinoma in situ	0	-	0	-					
	Invasive Carcinoma	2	0.3	0	-					

† p-value of the Odds Ratio and G-Test of Independence. ± p-value of the Logistic Regression. Variables were adjusted for each other within each group. Statistically significant values are denoted by an asterisk (*). A 95% Confidence Interval (CI) was calculated for these comparisons, and a significance level of *p* ≤ 0.05 was adopted for all analyses. Cramer’s V was used to assess the strength of association, categorized as weak (≤ 0.29), moderate (0.3–0.49), or strong (≥ 0.5)

## Discussion

This study demonstrated a high variability of high-risk oncogenic HPV genotypes in our population between 2013 and 2017 from several municipalities in the Brazilian Amazon that who spontaneously sought care at the health unit. Most of these women were asymptomatic and presented high socio-epidemiological vulnerability. The overall prevalence of HPV infection in our study was 13.84%. We observed a trend of increasing prevalence in the municipality of Irituia (21.25%, *p* = 0.0664), a rate like that observed among vulnerable young populations aged 25 to 29 years in low- and middle-income countries [3]. Health policies in Brazil are implemented through the Unified Health System (SUS), which offers comprehensive care through a universal, decentralized network organized at different levels of complexity in all regions of the country [25]. The main policy for addressing cervical cancer is the National Strategy for the Control and Elimination of Cervical Cancer (ENCECC), which since its creation in the late 1990s with the “Viva Mulher” program has been based on health education measures and screening using the Conventional Pap test for women aged 25 to 64, health education, and vaccination against quadrivalent HPV [26–28]. Despite good availability in urban and semi-urban environments, it faces challenges in consolidating itself in remote areas of the Amazon, due to long distances and the political and administrative challenges faced by local governments [12, 14, 29]. The impacts of this can be observed in official data on CC which show northern Brazil concentrating the highest rates in the country for consecutive years, raising questions about the significant challenges regarding the availability of public resources, geographical barriers, awareness campaigns to improve participation, quality control of the Pap tests among others. Our results show that the policy to combat CC may be insufficient to guarantee better results in the northern region, since only 21% of cases in Brazil are diagnosed in the early stages [30], with an incidence rate of 20.48 cases per 100,000 women and a mortality rate of 9.07 per 100,000 women [5, 29, 31, 32]. Our study population was entirely received in the capital due to the lack of these services in the interior of the Amazon, a common phenomenon in the Amazon where young people travel long distances to access basic health care in larger cities, overloading Basic Health Units (UBS) and hindering the decentralization policies promoted by the SUS since its creation [33]. The highest prevalences in the Amazon region are among young women, mostly underserved in terms of healthcare, with figures ranging from 15.5% to 63.3% in specific subpopulations such as people living with HIV, reflecting significant regional variability [19–21, 34, 35].

The Pap smear test, despite being low-cost and easy to perform, has low specificity and is not able to detect asymptomatic infections. Currently, Brazil has adopted a single-dose HPV vaccination scheme to improve vaccine acceptance and reduce hesitancy. Furthermore, HPV genotyping to identify oncogenic genotypes is now part of routine primary healthcare. Although promising for the early diagnosis and treatment of this infection, as well as for the long-term prevention of cervical cancer, these strategies are not yet available in all healthcare networks in the Amazon region [36, 37]. Despite identifying 63 samples positive for High-Risk Oncogenic HPV, there was a predominance of asymptomatic women, such that cytology was normal in 94.9% of cases. This reinforces the need for more accurate diagnostic methods for this infection in the asymptomatic population. In our study, women from eighteen Amazonian municipalities and women aged ≤ 25 years were three times more likely to acquire this infection (OR: 3.2902, *p* < 0.0001). Emerging social perceptions and risky sexual behaviors commonly observed in younger populations contribute to the vulnerability to sexually transmitted infections (STIs) [38]. A higher likelihood of acquiring HPV infection was observed in our study population, as unmarried women were nearly twice as likely to acquire HPV (OR: 1.9539, *p* < 0.0001), possibly due to their openness to new sexual experiences. Additionally, the lack of condom use in sexual relationships nearly doubled their chances of acquiring HPV (OR: 1.6563, *p* = 0.0032). Although the use of male condoms is questionable for HPV protection due to skin-to-skin contact in uncovered genital areas, co-infections with other STIs in women positive for high-risk HPV contribute to cervical carcinogenesis [39, 40]. Vulnerable female populations often exhibit risky sexual behaviors. This is particularly common among young populations residing in countries with limited screening and health education policies [41–44]. The study period coincided with the initiation of Brazil’s HPV vaccination program, therefore, we did not assess the vaccination status of participants. However, HPV vaccination faces significant gaps in adherence among peripheral, Amazonian, and school-aged populations, highlighting shortcomings in the management strategies for preventable diseases [23, 29, 45].

We observed that high-risk HPV genotypes were present in all municipalities with positive infection cases, representing more than half of the genotyped samples. Data on HPV genotypes in Brazil are limited to epidemiological studies, which confirm the high frequency of HPV16 and HPV18 genotypes associated with cervical lesions and tumors [46–49]. In the Amazon, the distribution of HPV genotypes predominantly includes these same genotypes, associated with cytological findings and cervical tumors [21, 50, 51]. The high variability of oncogenic HPV types has been documented since 2014 [22] while HPV genotype screening in Brazil only began in 2024 [10, 52] and is progressing slowly due to its high cost and continued restriction to urban centers, posing a significant barrier for women in remote communities. Globally, the distribution of HPV genotypes follow a specific pattern or concentrate within a well-defined social stratum. However, oncogenic HPV genotypes are found in up to 100% of cervical tumors in developing countries, with HPV16 being particularly prevalent in countries of the Southern Hemisphere [3, 6, 29]. Interestingly, despite the high prevalence and wide distribution of HPV16 and other oncogenic HPVs in this study, we observed no association with the cytological conditions of the participants. A considerable proportion of coinfections involving up to two HPV genotypes (14%, 8/57) and multiple infections with more than two HPV genotypes (22.8%, 13/57) were observed among the positive samples that demonstrated quality for genotyping, suggesting an epidemiological scenario with multiple distributions. Although we did not map transmission routes using molecular epidemiology, we believe that transmission chains remain sustained, maintaining subclinical characteristics likely due to an unvaccinated population [48, 53].

This study presents several limitations, the low quality of DNA obtained prevented us from genotyping all samples. One limitation is the lack of nucleic acid amplification techniques, which precluded any phylogenetic analysis. Additionally, the tests used in this study revealed a limited profile of HPV genotypes. The low sample size in some municipalities hindered more robust comparative analyses. Furthermore, there is a potential for response bias among study participants, who may have responded to questions about sexual behavior in accordance with socially accepted stigmas.

## Conclusion

The high variability of high-risk oncogenic HPV genotypes, particularly HPV16, coupled with the high likelihood of HPV infection among asymptomatic, young, unmarried women who do not use condoms during sexual intercourse in various municipalities of remote Amazonian regions, revealed significant exposure of our population to infectious risk to develop Cervical Cancer and other epidemiological vulnerabilities within this population. Women, lacking access to adequate diagnostic and subsequent followup and adequate treatment resources in their home communities, may be more susceptible to acquiring cervical cancer (CC). The findings of this study are crucial for evaluating the impact of policies aimed at addressing cervical cancer and can guide the implementation of preventive measures, as done recently as the HPV DNA diagnosis as the initial strategy to prevent CC. Further studies are needed to assess the prevalence and genotypic distribution of high-risk HPV and to strengthen public preventive strategies as the adoption of the nonavalent HPV vaccine to avoid cervical cancer in the Amazon region.

## Supplementary Information

Supplementary Material 1

Supplementary Material 2

Supplementary Material 3

## Data Availability

The data are under the custody of the researchers due to ethical considerations related to participant confidentiality and privacy.
