# Supplementary material for: High-risk oncogenic HPV genotypes in vulnerable women from the Amazon: a cross-sectional retrospective study
Source: Virol J. 2026 Jun 11;23:164. doi: 10.1186/s12985-026-03220-3 (PMC13295212; doi:10.1186/s12985-026-03220-3)
Supplement: Supplementary file 1 — Supplementary Material 1 [file 12985_2026_3220_MOESM1_ESM.docx]

**Supplementary material 3 (S2)**: Distribution of High-Risk Oncogenic HPV in Co-Infections and Multiple Infections Among Asymptomatic Young Women from the Amazon.

| Samples | HPV genotypes *† | Type of Infeccion |
| --- | --- | --- |
| 152 | **16**, 54, **59**, 62, 66, 73, 81 | Multiple |
| 235 | **16**, **45** | Co-infection |
| 269 | 6, **16**, CP6108, 59, 54 | Multiple |
| 313 | 6, **16**, **51**, 61 | Multiple |
| 388 | **16**, **35**, **56**, 61, 84 | Multiple |
| 443 | 67, **51**, **16** | Multiple |
| 519 | **16**, **33**, **51** | Multiple |
| 111 | **18**, 53 | Co-infection |
| 212 | **18**, **31**, **35**, **59** | Multiple |
| 225 | **18**, 53 | Co-infection |
| 153 | **31**, 61 | Co-infection |
| 468 | 6, **31**, 72, 83 | Multiple |
| 368 | **35**, **56**, 61, 84 | Multiple |
| 302 | **39**, **52**, 68 | Multiple |
| 275 | **56**, **59**, 70 | Multiple |
| 140 | **58**, 66, 81, CP6108 | Multiple |
| 266 | **58**, **59** | Co-infection |
| 618 | **58**, 61 | Co-infection |
| 165 | **59**, **73** | Co-infection |
| 170 | 73, 80, **59** | Multiple |
| 300 | **59**, 61 | Co-infection |
| * HPV genotypes identified from samples suitable for the Array Typing technique. †High-risk oncogenic HPV genotypes are highlighted in bold. | | |
