# Supplementary material for: High-risk oncogenic HPV genotypes in vulnerable women from the Amazon: a cross-sectional retrospective study
Source: Virol J. 2026 Jun 11;23:164. doi: 10.1186/s12985-026-03220-3 (PMC13295212; doi:10.1186/s12985-026-03220-3)
Supplement: Supplementary file 2 — Supplementary Material 2 [file 12985_2026_3220_MOESM2_ESM.docx]

**Supplementary Material 2:** Prevalence of HPV Infection genotypes in Municipalities of origin in the State of Pará, Brazilian Amazon.

| Municipalities | Total (n=1.279)^a^ | | HPV (+)  (N=182) | | *p-*value* | HPV total†  N=107 | | HPV genotypes | High-Risk HPV±  N=63 | | |
| --- | --- | --- | --- | --- | --- | --- | --- | --- | --- | --- | --- |
|  | N | % | N | % | 0.0664 | N | % |  | N | % | |
| Belém | 990 | 77.4 | 139 | 14.04 |  | 86 | 80.4 | **16 (n=11),** **18 (n=8),** **31 (n=2),** **33 (n=1),** **35 (n=2)**, **39 (n=1)**, 42 (n=1), **45 (n=1)**, **51 (n=4)**, **52 (n=1)**, 53 (n=5), 54 (n=2), 55 (n=1), **56 (n=2)**, **58 (n=7)**, **59 (n=7)**, 61 (n=6), 62 (n=2), 66 (n=3), 67 (n=1), 68 (n=1), 70 (n=1), 72 (n=1), **73 (n=2)**, 81 (n=3), **83 (n=1)**, 84 (n=1), CP6108 (n=3), 6 (n=4), 11(n=1). | 50 | 79.3 | |
| Ananindeua | 177 | 13.9 | 25 | 14.12 |  | 10 | 9.3 | **16 (n=1), 45 (n=1), 58 (n=1), 59 (n=3)**, 61 (n=1), 70 (n=2), 73 (n=1). | 6 | 9.5 | |
| Irituia | 80 | 6.2 | 17 | 21.25 |  | 10 | 9.3 | **16 (n=2), 31 (n=1), 33 (n=1), 51 (n=1)**, 54 (n=1), **58 (n=1)**, 61 (n=1), 66 (n=1), 72 (n=1). | 5 | 7.9 | |
| Marituba | 32 | 2.5 | 1 | 3.12 |  | 1 | 1 | **16 (n=1).** | 1 | 1.6 | |
| ^a^ Municipalities that reported at least one positive case. *The G-test of independence was calculated considering a 95% Confidence Interval (CI) and a significance level of p ≤ 0.05. †HPV types identified. ± High-risk HPV genotypes are highlighted in bold. | | | | | | | | | | |  |
