# Supplementary material for: High-risk oncogenic HPV genotypes in vulnerable women from the Amazon: a cross-sectional retrospective study
Source: Virol J. 2026 Jun 11;23:164. doi: 10.1186/s12985-026-03220-3 (PMC13295212; doi:10.1186/s12985-026-03220-3)

**Supplementary Material 1:** Distribution of HPV genotypes by municipality in the state of Pará, Amazon region of Brazil.


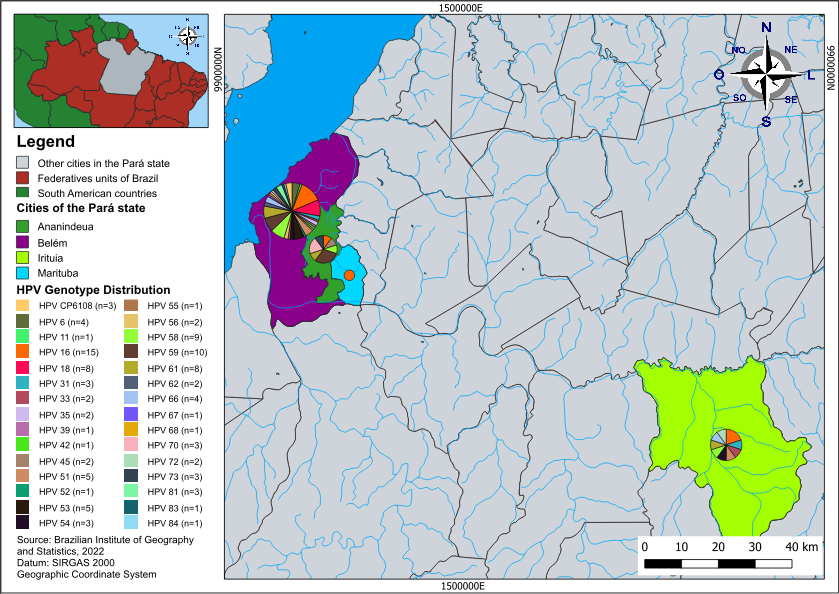

Supplement: Supplementary file 3 — Supplementary Material 3 [file 12985_2026_3220_MOESM3_ESM.docx]
